# Effect of Sterilization Process and Storage on the Antioxidative Properties of Runner Bean

**DOI:** 10.3390/molecules23061409

**Published:** 2018-06-11

**Authors:** Rafał Wołosiak, Beata Drużyńska, Małgorzata Piecyk, Ewa Majewska, Elwira Worobiej

**Affiliations:** Division of Food Quality Assessment, Faculty of Food Sciences, Warsaw University of Life Sciences–SGGW, 159 Nowoursynowska St., 02-787 Warsaw, Poland; beata_druzynska@sggw.pl (B.D.); malgorzata_piecyk@sggw.pl (M.P.); ewa_majewska1@sggw.pl (E.M.); elwira_worobiej@sggw.pl (E.W.)

**Keywords:** antioxidant activity, food processing, legumes, lipid oxidation, polyphenols, protein composition

## Abstract

In this study, we investigated the effect of standard preservation of bean seeds on changes in contents and activity of their selected components: dry matter, ash, different forms of nitrogen, composition of protein fractions; total phenolics and condensed tannins; ability to chelate iron(II) ions; antiradical activity against ABTS^•+^ and DPPH^•^; and capability for inhibiting autoxidation and enzymatic oxidation of linoleic acid. The conducted technological process caused various changes in contents of nitrogen forms and partial loss of phenolic compounds. The antiradical and antioxidative activity of the extracts decreased significantly, while an increase was observed in their ability to chelate Fe(II). These changes were due to the migration of active compounds to the brine, and to their structural transformations and degradation. Longer storage of the sterilized product caused restoration of part of the antiradical activity of the seeds.

## 1. Introduction

Leguminous seeds are important dietary constituents in many regions in the world. In developing countries, they are a basic source of proteins and other nutrients. Epidemiological surveys have demonstrated an association between the consumption of leguminous seeds and lowered risk of incidence of some diseases, such as cancers, type II diabetes, or cardiovascular diseases [1]. For this reason, the UN General Assembly has declared the year 2016 an International Year of Pulses with the aim to increase the world population’s awareness regarding their health-promoting properties. Health benefits stemming from the consumption of pulses are due to their multiple compounds which exhibit biological activity and antioxidative effects [2]. Many studies have proved a high antioxidative potential of such compounds as flavonoids, tannins, or phenolic acids [3]. They inactivate free radicals, form complexes with transition metals, and inhibit the action of lipoxygenases and other enzymes which catalyze oxidation reactions [3,4]. Some studies have revealed the ability of legume albumins (mainly of bean) to inactivate free radicals and inhibit the autoxidation process or enzymatically catalyzed oxidative processes. Similar properties were demonstrated for pea and broad bean globulins [5,6]. Such activities of food components are very important, as free radicals pose a serious threat to human health, because they are metabolized within the body and attack biologically active substances, such as proteins, fatty acids, and nucleic acids. This can lead to cell and tissue damage and, in consequence, to the development of many serious diseases [7]. In addition, free radicals accelerate the aging process [8]. Besides that, bean phenolics are suggested to exhibit other important characteristics for modern civilization bioactivities: anti-obesity and anti-inflammatory [9]. Beans are a source of other important compounds, sterols, tocopherols, and polyunsaturated fatty acids, which may be found in nutritionally significant levels, even after heat treatment [10].

Having reached technological maturity, legumes—which are significant sources of antioxidants but are seasonal vegetables—are subjected to various preservation treatments (freezing, sterilization) and consumed usually after technological or culinary processing. These processes might significantly influence the efficiency of their natural antioxidants [11], however, this influence in the case of legumes has not been widely investigated. Few studies were also devoted to runner bean of the genus *Phaseolus*, of which the most important and the most thoroughly scrutinized species is common bean (*Phaseolus vulgaris* L.), represented by more than 14,000 cultivars. Runner bean (*Phaseolus coccineus* L. syn. *Phaseolus multiflorus* Willd.) is a slightly less significant but popular crop; it is the second most common *Phaseolus* species in the world after common bean, popularly grown mostly in America and Europe [12,13]. Runner beans may be treated as an important source of fiber, minerals, and some vitamins for consumers, they are very rich in proteins and have high energetic value [14,15]. A dimeric lectin of MW 66 kDa found in *Phaseolus coccineus* seeds exhibited antiproliferative and cytokine-inducing activities with suggested therapeutic utility [16]. Research conducted by Worobiej et al. [17] also demonstrated differences in protein preparations made of common bean seeds and runner bean seeds, including over 2.5-fold higher presence of aromatic residues of amino acids on the surface of runner bean proteins, but a lower content of available thiol groups. Both factors may alter antioxidant activity of proteins. Considering the above, the objective of this study was to determine the effect of sterilization and storage on the composition and activities of antioxidative compounds of white runner bean.

## 2. Results

Raw seeds had a high content of ash (4.1%, Table 1) that is similar to, but slightly lower than, the values reported by Piecyk et al. [18] for two varieties of runner bean, i.e., 4.2% and 5.2%. In sterilized seeds, its content decreased significantly (by ca. 70%), which resulted from the migration of minerals to the brine during sterilization. This is consistent with findings from other studies with hydrothermally treated seeds [18,19,20,21]. The content of nitrogen reached 3.8% d.m. in raw seeds and increased by ca. 30% in sterilized seeds stored for 4 months. Storage for another 8 months caused the content of nitrogen compounds to decrease in the seeds but to increase in the brine.

The content of soluble nitrogen compounds in raw bean seeds reached 1.5%, which accounted for 40% of total nitrogen substances (Table 2). After sterilization, the content of soluble nitrogen in seeds decreased significantly as a result of migration to the brine. Such a decrease was also induced by storage of sterilized seeds, for a lower nitrogen content was found in seeds stored for 12 months compared to 4 months. This is also confirmed by an increasing content of nitrogen compounds in the brine during the longer storage period. Worthy of notice is that at the first stage of storage (4 months), mostly non-proteinaceous compounds migrated to the brine, because they constituted 58% of all nitrogen compounds of the brine, while during successive storage their content decreased to 31%.

**Figure 1:**
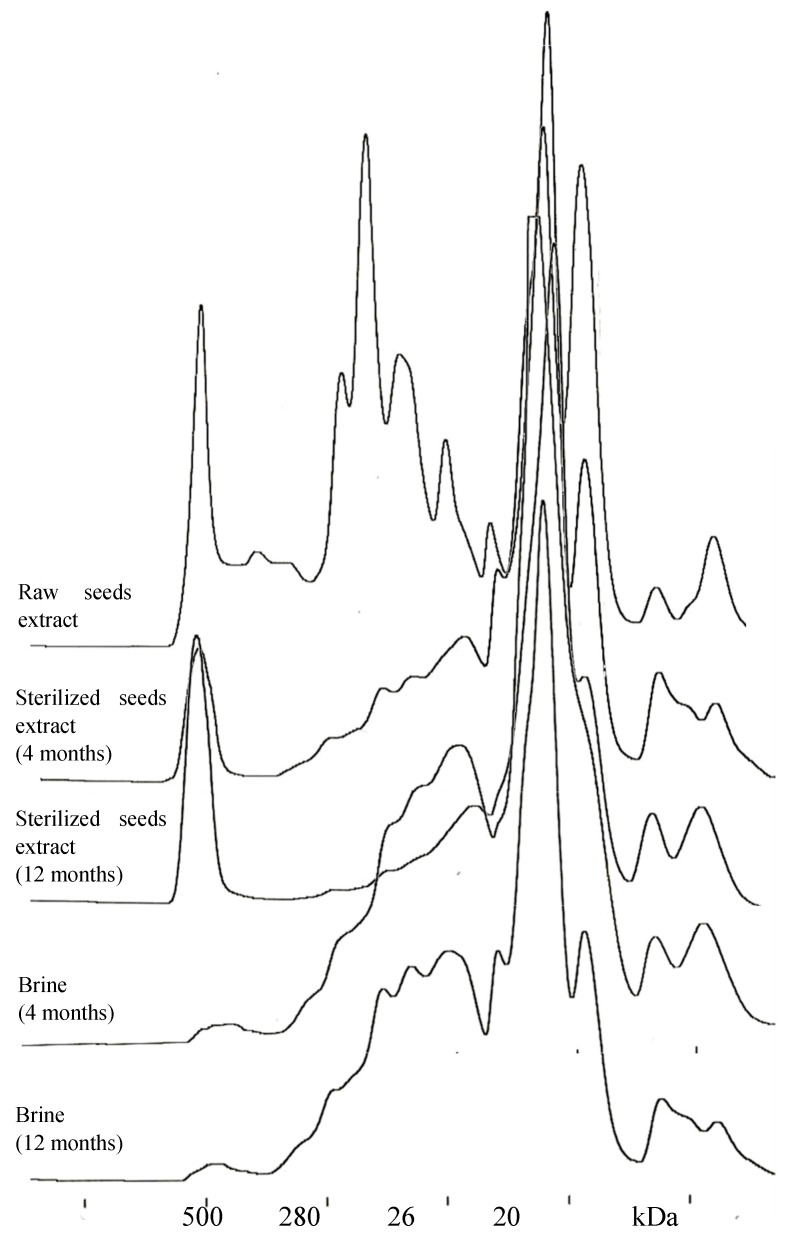
Gel filtration chromatograms of protein fractions in water extracts from white bean seeds before and after processing and in the brine.

Separations of aqueous extracts conducted using SE-HPLC demonstrated changes in proteins induced by the sterilization process and storage in both seeds and brine (Figure 1). The extract from raw seed analyses demonstrated significant amounts of globulin 7S (180 kDa) and its subunit (50 kDa) [22,23]. Molecular weight of 7S proteins ranges from 150 to 200 kDa, depending on legume species [24]. High contents were also found in the case of the fraction with the molecular weight of 100 kDa (most likely albumin) and polymeric fractions with molecular weights exceeding 500 kDa. The 7S fraction was not detected in extracts prepared from sterilized seeds nor in the brine. Presumably, sterilization caused the formation of insoluble aggregates of the main storage protein in bean seeds. This resulted in a reduced solubility of nitrogen observed in our study. In turn, soluble fractions with a high molecular weight (>500 kDa) found in extracts from raw and sterilized seeds occurred in low amounts in the brine, which contained mainly fractions characterized by high solubility in water, corresponding to albumins. All extracts and brine showed a high content of the fractions with molecular weight <20 kDa, which confirms the high content of non-proteinaceous nitrogen compounds in their samples (Table 2).

The total content of polyphenols (TPC) accounted for 1.6 g/100 g d.m. in raw bean seeds (Table 3). Similar content in white beans was reported by Akond et al. [3]. The sterilization process caused a statistically significant decrease in TPC after both 4 and 12 months of storage (by 30% and 46%, respectively). The highest content of condensed tannins was also detected in raw seeds and was observed to decrease after processing. After 12 months of storage, the content of tannins decreased by 30%. Other authors also observed a decrease in the content of phenolics in heat-treated beans [25]. Decreases noted during storage in contents of both total polyphenols and condensed tannins were most likely due to the migration of water-soluble forms of phenolic compounds to the brine. Similar observations were also made by Parmar et al. [26]. The lowered content of polyphenols may also result from their degradation under the influence of high temperature [27]. Some research demonstrated transformations of polyphenols upon the high temperature and pressure treatments, which led to their polymerization and ultimately impaired their extraction [28]. Such changes were confirmed in our study in the case of condensed tannins, because their content in beans was consistently lower, but in the brine reached only 0.01% after both storage periods of sterilized seeds.

In the case of both aqueous and 70% acetone extracts, the process of sterilization caused an increase in iron(II)-chelating ability (Table 2 and Table 3). In aqueous extracts, the increase in chelating ability was from less than 2 µM Fe/g d.m. to around 6 µM Fe/g d.m., whereas, in 70% acetone extracts, the increase was to around 5.5 µM Fe/g d.m. A similar tendency was observed in the study conducted by Wołosiak et al. [29] for sterilized seeds of green pea and string bean. Changes in the chelating ability of proteins isolated from legumes subjected to heat treatments were also demonstrated by Arcan and Yemenicioğlu [30], however, direction of these changes depended on seed type. The increase in iron(II) ion-chelating ability in the aqueous extracts may be due to multi-oriented changes in proteins, e.g., changes in contents of available amino acids. Studies with other pulses demonstrated greater increases in leucine, lysine, and proline contents in protein of sterilized seeds than of frozen or cooked seeds [31].

Aqueous extracts prepared from raw bean seeds and from treated bean seeds showed a significantly higher antiradical activity against ABTS^•+^ compared to the 70% acetone extracts (Table 4). The same dependency was confirmed in studies with other legume seeds, i.e., string bean and green pea [29], whereas an opposite one was found for broad been seeds [32]. Processing caused a highly significant decrease in the activity of antioxidants extracted with water and 70% acetone against ABTS^•+^ (even ca. 7-fold and 6-fold decrease, respectively, considering the total mass of the material). The extracts from sterilized been seeds stored for 12 months were characterized by a slightly higher activity than the extracts prepared from the same material stored for 4 months, despite simultaneous increase of the activity in the brine. This indicates that extracts’ ability to inactive ABTS^•+^ was influenced by changes of antioxidants induced by storage after sterilization to a larger extent than by their successive migration to the brine. The final activity of brine compounds was, however, similar to the activity of aqueous extracts and exceeded (by ca. 35%) the activity of 70% acetone extracts. On the other hand, considering the contribution of the brine and seeds in the obtained product, it may be concluded that ca. 80% of its activity against ABTS radical cations remained in the seeds.

Changes in the activity of the analyzed extracts against ABTS^•+^, expressed per dry matter (the content of which decreased 3-fold), were obviously different (lower) than those in the activity expressed per total weight of the product after sterilization. After 12 months of bean storage, ca. 2-fold decrease was observed in the case of the aqueous extracts and ca. 1.5-fold decrease in the case of 70% acetone extracts. In turn, analyses demonstrated a significantly higher effectiveness of brine components compared to components of both types of extracts, which indicates that mainly compounds with a high antioxidative potential were migrating to the brine. This migration may be the reason for their significant losses in products of this type.

Deterioration of the antiradical activity of 70% acetone extracts from pulses (cowpea, green pea, and chickpea) under the influence of high temperature treatments (i.e., drying, autoclaving) was also demonstrated by Siddhuraju and Becker [33] and Nithiyanantham et al. [34], however, a higher activity was maintained by the “dry-treated” samples. A significant decrease in the ability to inactivate ABTS radical cations was also demonstrated in the case of 70% acetone and aqueous extracts from sterilized seeds of green pea and string bean [29].

In contrast to the aforementioned experiment, a 10-fold higher activity against DPPH radicals was shown for the compounds extracted from the experimental material using 70% acetone than using water (Table 5). However, after treatment and storage, their activity decreased significantly (over 70 times), whereas the activity of antioxidants extracted with water decreased only ca. 8-fold. This phenomenon resulted in a comparable activity of both types of extracts after seed preservation and storage. In this case, the activity determined in the brine significantly exceeded the activity of antioxidants remaining in the seeds that were extracted with water, regardless of storage time, and was more similar to the activity of compounds which remained in the seeds after treatment and storage and were extracted with a solution of 70% acetone. However, taking into account the contribution of seeds and brine in the manufactured product, it may be observed that the activity of the brine constitutes ca. half the total activity of the preserved seeds.

Similar changes in the activity against DPPH radicals were demonstrated by Xu and Chang [35] after traditional and pressure cooking of chickpea. They noted a few-fold decrease in the activity of cooked seeds and similar activity to that of the final product detected in water. These changes were similar, despite various conditions of treatment processes. Slightly lesser changes could be observed during analogous processing of yellow pea and green pea (50–70% loss of seed activity and a slightly lower activity of the brine). The lack of such significant changes in the activity in the above-cited study concerned only lentil, however, these authors applied a significantly shorter hydrothermal treatment. These observations point to a similar character of changes in the antiradical activity of various legume seeds and are consistent with results obtained in our work.

Considering dry matter of seeds and brine, the initial activity of compounds extracted from seeds with water decreased 3-fold as a result of sterilization and storage of the sterilized product for 4 months. The activity which appeared in the brine was incomparably stronger than the activity of compounds extracted from seeds using water and 70% acetone. This again points to selective migration of compounds with a high antioxidative activity to the brine. Successive increase was noted in the activity detected in the brine during storage, whereas no significant changes were further observed in the activity of seed components.

The process of sterilization also caused deterioration of the extracts’ ability to inhibit the enzymatic oxidation of linoleic acid, which was due to both rehydration of seeds and changes in antioxidants upon hydrothermal treatment and storage (Table 6). In the autoxidation reaction of a linoleic acid emulsion, a significant activity was exhibited only by the aqueous extract prepared from raw seeds (36%). The antioxidative activity of compounds soluble in the acetone solution was negligible, likewise for the activity of water-soluble compounds determined in the sterilized product. Only the activity determined in the brine reached higher values, as it increased 3-fold as a result of prolonged storage. In contrast to the non-enzymatically catalyzed experiment, in the reaction catalyzed with lipoxygenase, the aqueous extract demonstrated a 2-fold lower antioxidative activity compared to the 70% acetone extract. Many studies have confirmed strong antioxidative properties of polyphenols, not only regarding their capabilities for inactivating free radicals and chelating ions of transition metals, but also inhibiting oxidases [2]. The antioxidative activity of the 70% acetone extract was also relatively good after seed sterilization and significantly exceeded that of the aqueous extract. However, it decreased during storage (in contrast to the activity determined in the autoxidation process).

The statistical analysis demonstrated quite weak correlation between activity against ABTS radicals and peroxides of linoleic acid (r = 0.889, α = 0.05) and between the activity against stable DPPH radicals and activity in the reaction catalyzed by lipoxygenase (r = 0.748, α = 0.05). No significant correlations were determined when analyzing the above activities as expressed per dry matter of the product. While referring to activities obtained from contents of product components, a correlation was only found between the activity against DPPH radicals in aqueous extracts and soluble nitrogen content in these extracts (data expressed per dry matter of the product: r = 0.996, α = 0.001). Poor and few correlations confirm the above-noted fact of various behaviors of antioxidants extracted from raw and sterilized bean seeds in different experimental systems.

## 3. Materials and Methods

### 3.1. Plant Material

White beans (*Phaseolus coccineus*) cv. Jaś Karłowy of proper technological maturity (dry seeds) were obtained from Florpak (Warsaw, Poland). Seeds were packed into 400 g cans. Solution of 1.3% NaCl was used as brine and the ratio between seeds and solution was 6:4 (*w*:*w*). Seeds were rehydrated (30 min, 96 °C) and then sterilization was done in the autoclave Rotorzwerg (Stock, Neumünster, Germany) until the temperature in geometrical center reached 123 °C. Temperatures of heating medium and the contents of the cans were measured with SSA 12,050 G 7000 TS thermometers connected to a four-channel recorder CTF 9004 (Ellab, Hillerød, Denmark). The investigations were performed on raw and sterilized seeds after 4 and 12 months of storage.

### 3.2. Material Preparation

Raw or sterilized material was homogenized in a laboratory knife grinder (Grindomix GM 200, Retsch, Haan, Germany). Extracts in water and 70% (aqueous) acetone were prepared at material to solvent ratio of 1:10 by shaking for 2 h at ambient temperature and then centrifuging samples (10,000 rpm, 20 min).

### 3.3. Chemical Composition

Basic composition was determined in homogenates by means of AOAC methods [36]. Total, soluble, and non-protein nitrogen (after protein precipitation using trichloroacetic acid to final concentration of 12.5%) were analyzed by Kjeldahl’s method. All analyses were performed in four replicates except for ash content (five replicates).

### 3.4. Determination of Condensed Tannins Content (CTC)

The condensed tannins were analyzed by using spectrophotometric method with vanillin reagent described by Price’a et al. [37]. (+)Catechin (Sigma, St. Louis, MO, USA) was used as a standard in this experiment. The content of condensed tannins was expressed as g of catechin equivalents (CAE)/100 g d.m. Analyses were performed in four replicates.

### 3.5. Determination of Total Polyphenols Content (TPC)

The amount of total polyphenols in 70% acetone extracts was determined with the Folin–Ciocalteu’s reagent [34,38]. Absorption at 700 nm was measured (Shimadzu UV-160A). The content of total polyphenols was expressed as gallic acid (GA, Sigma) equivalents in g GAE/100 g d.m. Analyses were performed in four replicates.

### 3.6. Size-Exclusion HPLC

Chromatographic analyses were performed using a pump system (model LC-6A, Shimadzu, Kyoto, Japan) equipped in a manual sample injection valve (Rheodyne, Cotati, CA, USA) and a spectral UV detector (Shimadzu, Kyoto, Japan). Water extracts were injected onto Supradex 200 HR analytical column (10 mm × 30 cm, Amersham Pharmacia Inc., Piscataway, NJ, USA) and detected at 280 nm. The column was equilibrated in elution buffer (50 mm phosphate buffer containing 150 mm NaCl, pH 7.0) at a flow rate of 0.4 mL/min and calibrated using a set of protein standards from Pierce (12,500–540,000 Da). Molecular weights of the fractions in extracts were determined using the equation of Andrews [39]. Analyses were performed in two replicates.

### 3.7. Chelating Ability

The ability of aqueous and 70% acetone extracts to chelate Fe(II) ions was determined spectrophotometrically using FeCl_2_ and ferrozine (3-[2-pyridyl]-5,6-diphenyl-1,2,4-triazine-4′,4′′-disulfonic acid sodium salt, Sigma) as described by Lai et al. [40]. The results were calculated based on the amount of iron added to the samples and using a calibration curve prepared from FeCl_2_ solutions. Chelated Fe(II) was expressed in µmol/g d.m. Analyses were performed in six replicates.

### 3.8. Antiradical and Antioxidant Activity

Antiradical activity of the extracts was determined towards ABTS^•+^ radical cations [41] and DPPH^•^ stable radicals [42] and the results were calculated basing on Trolox standard curve. Antioxidant activity was also measured in linoleic acid emulsion during hemoglobin-catalyzed autoxidation and enzymatic reaction catalyzed by lipoxygenase. In both cases, the amount of hydroperoxides was measured applying the spectrophotometric (λ = 480 nm) ferric thiocyanate method [43]. The results were expressed as percent of oxidation inhibition relating to control. Analyses were performed in four replicates.

### 3.9. Statistical Analysis

Results obtained were expressed as means and standard deviation and were subjected to statistical analysis (Statistica v. 10.0 software, Dell Inc., Tulsa, OK, USA), including ANOVA with post hoc Duncan test at *p* < 0.05 for the significance of differences and simple linear regression.

## 4. Conclusions

We observed a significant decrease in the contents of the analyzed dry matter components soluble in water (nitrogen substances) and in 70% acetone (phenolic compounds), resulting from seed rehydration, migration of the analyzed compounds to the brine, and their structural or degradation changes. Lack of statistically significant correlations between the activity of extracts and contents of the analyzed groups of compounds confirms selective division of antioxidants in sterilized seeds and the brine.

Components of the aqueous extracts from bean seeds were more effective in inactivation of ABTS^•+^ and in inhibiting autoxidation processes of linoleic acid, whereas components soluble in acetone solution proved better in inactivating DPPH^•^ and inhibiting lipoxygenase action. Despite the observed loss of active compounds to the brine (being especially significant considering compounds active against DPPH^•^), their transformations resulting from product storage may be found beneficial in the case of activity against ABTS^•+^, and adverse considering activity against DPPH^•^. Processing and storage caused loss of activity against enzymatically and non-enzymatically oxidized linoleic acid, contrary to the capability of the extracted compounds for chelating the pro-oxidative iron(II) ions.

Migration of compounds with high antiradical activity to the brine may significantly diminish bioavailability of active compounds because the brine is usually discarded before consumption of canned products. However, considering the preponderance of the total activity remained in the preserved seeds over brine activity, and the necessity of hydrothermal treatment of dry seeds before consumption, the process of sterilization of runner bean seeds may be deemed a good preservation method, leading to creation of a convenient food product.

## Figures and Tables

**Table 1:** Characterization of raw and processed material (nd—not determined).

Sample		Total N g/100 g d.m.	Ash g/100 g d.m.	Dry Matter g/100 g
Raw seeds		3.8 ± 0.1 ^b^	4.12 ± 0.01 ^b^	89.2 ± 0.1 ^e^
Stored 4 months	sterilized seeds	5.0 ± 0.1 ^c^	1.29 ± 0.04 ^a^	30.2 ± 0.3 ^c^
	brine	2.9 ± 0.3 ^a^	nd	7.8 ± 0.1 ^b^
Stored 12 months	sterilized seeds	3.7 ± 0.1 ^b^	1.28 ± 0.01 ^a^	32.3 ± 0.1 ^d^
	brine	3.6 ± 0.1 ^b^	nd	6.7 ± 0.2 ^a^

Values are mean ± SD. Data with the same superscript alphabets in columns are not significantly different.

**Table 2:** Soluble and non-protein nitrogen content and Fe(II)-chelating ability of water extracts (nd—not determined).

Sample		Soluble N g/100 g d.m.	Non-Protein N g/100 g d.m.	Chelating Ability µmol Fe/g d.m.
Raw seeds		1.52 ± 0.02 ^c^	0.38 ± 0.0 ^b^	1.94 ± 0.02 ^a^
Stored 4 months	sterilized seeds	0.64 ± 0.01 ^b^	0.61 ± 0.0 ^c^	5.84 ± 0.01 ^c^
	brine	nd	1.67 ± 0.0 ^e^	2.17 ± 0.03 ^b^
Stored 12 months	sterilized seeds	0.26 ± 0.01 ^a^	0.17 ± 0.0 ^a^	6.04 ± 0.03 ^c^
	brine	nd	1.11 ± 0.0 ^d^	5.98 ± 0.01 ^c^

Values are mean ± SD. Data with the same superscript alphabets in columns are not significantly different.

**Table 3:** Contents of total polyphenols (TPC), condensed tannins (CTC), and Fe(II)-chelating ability in 70% acetone extracts (nd—not determined).

Sample		Total Polyphenols g GAE/100 g d.m.	Condensed Tannins g CAE/100 g d.m.	Chelating Ability µmol Fe/g d.m.
Raw seeds		1.62 ± 1.54 ^c^	0.65 ± 1.21 ^d^	1.88 ± 0.03 ^a^
Stored 4 months	sterilized seeds	1.14 ± 1.87 ^b^	0.56 ± 1.26 ^c^	5.30 ± 0.06 ^b^
	brine	nd	0.01 ± 0.01 ^a^	nd
Stored 12 months	sterilized seeds	0.88 ± 1.45 ^a^	0.46 ± 1.14 ^b^	5.73 ± 0.09 ^b^
	brine	nd	0.01 ± 0.01 ^a^	nd

Values are mean ± SD. Data with the same superscript alphabets in columns are not significantly different.

**Table 4:** Antiradical activities of bean seed extracts and brine against ABTS^•+^ (nd—not determined).

Sample		Water Extract		70% Acetone Extract	
		mg Trolox/100 g	mg Trolox/100 g d.m.	mg Trolox/100 g	mg Trolox/100 g d.m.
Raw seeds		355.4 ± 3.7 ^e^	398.4 ± 4.2 ^d^	192.0 ± 1.2 ^c^	215.3 ± 1.4 ^c^
Stored 4 months	sterilized seeds	50.9 ± 1.8 ^b^	168.7 ± 6.1 ^a^	33.9 ± 4.1 ^a^	112.3 ± 13.4 ^a^
	brine	23.7 ± 0.4 ^a^	304.4 ± 5.7 ^c^	nd	nd
Stored 12 months	sterilized seeds	65.7 ± 3.3 ^d^	203.4 ± 10.1 ^b^	44.7 ± 1.4 ^b^	138.3 ± 4.2 ^b^
	brine	61.0 ± 0.6 ^c^	910.5 ± 8.2 ^e^	nd	nd

Values are mean ± SD. Data with the same superscript alphabets in columns are not significantly different.

**Table 5:** Antiradical activities of bean seed extracts and brine against DPPH^•^ (nd—not determined).

Sample		Water Extract		70% Acetone Extract	
		mg Trolox /100 g	mg Trolox/100 g d.m.	mg Trolox/100 g	mg Trolox/100 g d.m.
Raw seeds		38.0 ± 0.1 ^e^	42.7 ± 0.5 ^b^	402.9 ± 27.1 ^b^	451.7 ± 30.3 ^b^
Stored 4 months	sterilized seeds	4.6 ± 0.1 ^b^	15.3 ± 0.2 ^a^	5.8 ± 0.1 ^a^	19.2 ± 0.2 ^a^
	brine	7.1 ± 0.2 ^c^	91.0 ± 2.1 ^c^	nd	nd
Stored 12 months	sterilized seeds	4.0 ± 0.2 ^a^	12.5 ± 0.7 ^a^	6.1 ± 0.2 ^a^	19.0 ± 0.7 ^a^
	brine	7.6 ± 0.4 ^d^	112.8 ± 5.9 ^d^	nd	nd

Values are mean ± SD. Data with the same superscript alphabets in columns are not significantly different.

**Table 6:** Antioxidant activities (%) of the extracts investigated against linoleic acid autoxidation (LOOH) and lipoxygenase-catalyzed oxidation (LOX); (nd—not determined).

Sample		Water Extract		70% Acetone Extract	
		LOOH	LOX	LOOH	LOX
Raw seeds		36.6 ± 0.8 ^c^	17.0 ± 4.5 ^c^	5.3 ± 1.0 ^b^	32.2 ± 9.7 ^b^
Stored 4 months	sterilized seeds	4.8 ± 0.4 ^a^	6.8 ± 2.1 ^b^	1.6 ± 1.4 ^a^	23.2 ± 7.1 ^b^
	brine	3.5 ± 1.6 ^a^	0.8 ± 2.7 ^a^	nd	nd
Stored 12 months	sterilized seeds	3.9 ± 3.1 ^a^	1.4 ± 2.0 ^a^	4.8 ± 1.4 ^b^	8.9 ± 3.5 ^a^
	brine	10.1 ± 3.2 ^b^	3.3 ± 1.9 ^a,b^	nd	nd

Values are mean ± SD. Data with the same superscript alphabets in columns are not significantly different.
